# Breeding custom‐designed crops for improved drought adaptation

**DOI:** 10.1002/ggn2.202100017

**Published:** 2021-09-20

**Authors:** Rajeev K. Varshney, Rutwik Barmukh, Manish Roorkiwal, Yiping Qi, Jana Kholova, Roberto Tuberosa, Matthew P. Reynolds, Francois Tardieu, Kadambot H. M. Siddique

**Affiliations:** ^1^ Centre of Excellence in Genomics and Systems Biology International Crops Research Institute for the Semi‐Arid Tropics (ICRISAT) Hyderabad India; ^2^ State Agricultural Biotechnology Centre, Centre for Crop and Food Innovation Murdoch University Murdoch Western Australia Australia; ^3^ Department of Plant Science and Landscape Architecture University of Maryland College Park Maryland USA; ^4^ Institute for Bioscience and Biotechnology Research University of Maryland Rockville Maryland USA; ^5^ Crop Physiology and Modelling International Crops Research Institute for the Semi‐Arid Tropics (ICRISAT) Hyderabad India; ^6^ Department of Agricultural and Food Sciences University of Bologna Bologna Italy; ^7^ International Maize and Wheat Improvement Center (CIMMYT) Texcoco Mexico; ^8^ Université de Montpellier, INRAE, Laboratoire d'Ecophysiologie des Plantes sous Stress, Environnementaux Montpellier France; ^9^ The UWA Institute of Agriculture The University of Western Australia Perth Western Australia Australia

**Keywords:** context‐dependent optimization, drought physiology, genome editing, genomic breeding, root system architecture, speed breeding

## Abstract

The current pace of crop improvement is inadequate to feed the burgeoning human population by 2050. Higher, more stable, and sustainable crop production is required against a backdrop of drought stress, which causes significant losses in crop yields. Tailoring crops for drought adaptation may hold the key to address these challenges and provide resilient production systems for future harvests. Understanding the genetic and molecular landscape of the functionality of alleles associated with adaptive traits will make designer crop breeding the prospective approach for crop improvement. Here, we highlight the potential of genomics technologies combined with crop physiology for high‐throughput identification of the genetic architecture of key drought‐adaptive traits and explore innovative genomic breeding strategies for designing future crops.

## Graphical abstract

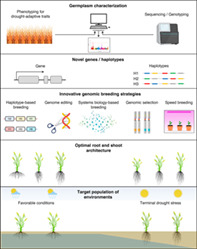

## 1 INTRODUCTION

Crop production must increase to meet the dietary needs of the global human population; however, this task is challenged by the fluctuating environmental conditions. The changing climate, referred to as “climate crisis,” is heading us toward a warmer and drier Earth.^1^ In the last decade, global economic losses in agriculture stemming from drought totaled approximately US $29 billion.^2^ It is anticipated that water demand for agriculture could increase two fold by 2050, with freshwater availability decreasing by up to 50% due to increasing climatic variations. Food security necessitates urgent investments in this domain, particularly for the development of high‐yielding crops that are climate‐resilient and more effective and/or efficient in using water than their prevailing counterparts.^3^

Conventional breeding programs have made impressive progress in the development of crop varieties adapted to drought conditions.^4^, ^5^, ^6^ However, this labor‐intensive process often takes many years to advance from the preliminary stages of assessing phenotypes and genotypes to initial crosses into commercial varieties. Conventional breeding ignores the genetic variability of adaptive traits that underlie yield, at the risk of (indirectly) selecting only those alleles that are beneficial in all tested environments.^7^ The alleles thus selected are infrequent compared with those alleles whose effects are context‐dependent.^8^ By contrast, context‐dependent optimization of traits has the potential to maximize positive effects on yield under specific environmental conditions. Therefore, to improve the production of adapted varieties, future breeding programs must combine desirable plant traits that complement climate, soil, and management practices (eg, sowing dates, fertilization, plant density, etc.) in target production systems.

Plant genomics plays a key role in improving crops, advancing environmental resilience and productivity.^9^ Technical innovations in applied genomics coupled with the availability of large‐scale sequencing data provide us with the capabilities for identifying genetic variation that underlies increasing crop performance and improving the efficiency of breeding.^10^, ^11^, ^12^ Furthermore, biotechnological approaches, including targeted genome editing using CRISPR‐Cas technologies, have expedited advances in the temporal and spatial regulation of genes and major pathways for drought adaptation.^13^, ^14^ A comprehensive understanding of the adaptive mechanisms under distinct drought scenarios is crucial for securing future harvests and fuelling the necessary genetic gain in crop improvement. Such gains can be driven by genetic variability and deployed by genomic breeding, more precise genetic modifications, and tailored management practices.

In this review, we start by discussing the major advances in crop leaf and root research associated with drought adaptation, and then describe how the context‐dependent optimization of above‐ and below‐ground traits offers opportunities to improve future crops. We discuss recent innovations in genomic breeding approaches that empower design‐based crop improvement, including haplotype‐based breeding, genome editing, systems biology, and genomic selection. Finally, we explore how speed breeding could interact with new‐age genomic breeding technologies to speed‐up crop development. Our goal is to provide a comprehensive overview of the processes related to drought and highlight possible ways to develop future crops in the face of increasing climatic fluctuations.

## 2 PHYSIOLOGICAL ADAPTATIONS TO DROUGHT

In times of drought, crops dynamically manage their water balance by: (a) limiting water loss by reducing leaf area and stomatal conductance^15^, ^16^; (b) enhancing soil water uptake by altering root growth and architecture^17^, ^18^; and (c) osmotic adjustment (OA), via accumulation of solutes in the cells.^19^ Here, we discuss some of the key adaptive traits in further detail.

### 2.1 Limiting water loss

Crops exposed to soil water deficit need to preserve available water by limiting transpiration while, in parallel, fixing adequate carbon to meet energy demands. Reduction in leaf area and stomatal conductance display a rapid response against dehydration by limiting the transpiration rate, thus budgeting soil moisture and maintaining increased leaf water potential levels. For instance, drought adaptation of stay‐green (*Stg*) sorghum is linked to reduced green leaf area at anthesis, lower tillering, and smaller upper leaves.^15^ These mechanisms facilitate remarkable plasticity to the crop for modulating canopy development in response to the intensity of drought stress. Furthermore, reduced stomatal conductance and transpiration rate, leading to better water‐use efficiency, enable increased drought adaptation in wheat.^20^ Low stomatal density also improves drought tolerance and water conservation properties in rice^16^ and barley.^21^ Leaf growth is mainly determined by vapor pressure deficit and available soil moisture, with a large genetic variability in the sensitivity to both conditions. As a result, leaf area evaluated at any particular time point is a consequence of the prevailing environmental conditions and genotype‐dependent sensitivity. Canopy development traits are mainly driven by leaf area,^15^ which is affected by other factors such as tillering or phyllochron, all of which depend on drought severity with genotype‐dependent sensitivities. Therefore, if the phenotypes associated with leaf area are to be included in broader research and breeding programs, precise and dynamic measurement of leaf area becomes essential. Phenotyping systems are being developed that strategically target leaf traits which are crucial and relatively easy to measure at the single‐leaf or whole‐canopy levels, and in some cases by remote sensing with drones.^22^, ^23^

### 2.2 Enhancing water uptake

The root system is the interface for soil water and nutrient acquisition, and physically anchors the plant to the soil substrate. In a plant root system, the coarse (or tap) roots play a role in plant anchorage and usually establish root system architecture, regulate rooting depth, and the capability of the plant to grow in dense soil layers. In contrast, fine (or lateral) roots are actively involved in water uptake, and mostly comprise of the length and surface area of the root system.^24^ Root architecture and its capability to acclimate in response to environmental fluctuations are key factors determining overall plant robustness.^18^, ^25^, ^26^ During soil water deficit, root systems change structurally to improve water and nutrient uptake from the soil profile. For instance, in soil environments with heterogeneous moisture distribution, roots can demonstrate hydro‐patterning, with a preference for lateral root emergence toward soil zones with higher water content, a process facilitated by auxin signaling.^27^ Using maize roots, it is experimentally demonstrated that growth is essential for perception of water availability to pattern lateral roots in plants.^17^ Hydrotropism represents another adaptive root response, where root tips propagate toward soil patches containing higher moisture content to optimize water procurement.^28^ Furthermore, root respiration provides the energy for root growth and maintenance, absorption of water molecules and ions followed by their transport into the xylem, highlighting the root physiological metabolic capability. A decline in root respiration and root biomass under severe water deficit is associated with improved grain yields and high drought adaptation in wheat cultivars.^29^ Root system architecture is becoming a key target for crop improvement; however, progress in this domain has been fairly slow, partly owing to challenges with efficient phenotyping of roots.^30^, ^31^ From a breeding viewpoint, more effective phenotyping approaches, which can evaluate large mapping populations or germplasm lines for proxies in the field and genetic variability of root characteristics on phenotyping platforms, are required to incorporate root traits in crop improvement programs.

### 2.3 Osmotic adjustment

OA is a metabolic process that plays a key role in drought adaptation through turgor maintenance and the protection of defined cellular functions by intercellular solutes.^19^ OA has been implicated in supporting crop yield under drought conditions. For instance, high OA wheat cultivars maintained better growth and yield, both of which were linked to enhanced leaf water potential relative to low OA cultivars.^32^ Mahmood et al.^33^ measured OA in 30 wheat genotypes subjected to well‐watered and drought stress conditions in the field. Here, OA was positively associated with kernel weight that directly contributed to yield, suggesting that wheat achieves OA to uptake more soil water during low water potential. A significant and positive correlation was observed between yield and OA capacity under terminal drought stress conditions in barley.^34^ Further, Moinuddin and Imas^35^ evaluated eight chickpea varieties for OA and specific osmolytes such as sugars, proline, nitrogen, and potassium. The contribution of the osmolytes to OA became more crucial with an increase in water deficit toward the reproductive stage. Here, grain yield showed a linear and positive correlation with high OA and relative water content under water deficit. The importance of OA as a preferable selection target from a breeding viewpoint has been a continuing trickle of skepticism. This is mainly due to the belief that drought‐adaptive genotypes with a better ability to adjust osmotically are typically characterized by slow growth and limited biomass production, because of metabolic needs for osmolyte biosynthesis. Under severe drought stress, increased accumulation of osmolytes may help crops withstand a prolonged drought episode and go through a more prompt and complete recovery after rehydration.^32^ OA by maintaining turgor in wheat exposed to slow drying soil, helps to partially sustain stomatal conductance, photosynthesis, and dry biomass accumulation at low levels of leaf water potentials.^36^ Importantly, the trade‐off between metabolic costs associated with OA and the potential advantages to the crop differs on a case‐to‐case basis as a function of the genotype, and the dynamics and intensity of drought scenarios.

## 3 TRAIT‐BASED BREEDING FOR DROUGHT ADAPTATION

Seed yield is usually the selection criterion when breeding crops for drought adaptation. However, yield is a complex and final‐stage trait, which is influenced by the environmental interaction with growth and development processes that occur throughout the crop cycle. A conventional breeding strategy aimed at improving performance under drought by selecting genotypes merely based on higher absolute yields is predicted to fall short of meeting future crop production demands.^37^ Limited genetic variation in yield among improved cultivars, high genotype × environment × management (G × E × M) interactions, and low heritability are some critical factors that could restrict future crop improvement efforts through direct selection for yield.^38^ By contrast, the genetic improvement of adaptive traits (such as biomass, harvest index, canopy temperature, etc.) through changes in leaf and/or root ideotypes hold enormous potential to increase productivity and genetic gain under drought conditions, as demonstrated in wheat.^39^ This is because such traits are genetically more variable among present‐day cultivars and have not been the target of conventional breeding efforts.^40^ Designing a crop with better adaptation to drought conditions demands a major effort in improving such adaptive traits. Therefore, a trait‐based strategy that evaluates genotypes on the basis of physiological responses to water deficit at the initial stages of plant growth could be more targeted to drought and time efficient.

## 4 CONTEXT‐DEPENDENT OPTIMIZATION PROBLEM

Many drought‐adaptive traits often possess a twofold effect: positive in severe terminal stress conditions and negative in favorable (or milder drought stress) conditions, or vice versa.^7^ Remarkable results obtained in one drought scenario might confer only limited productivity gains in other geographical areas experiencing water deficit. Moreover, genetic trade‐off among adaptive traits may also occur. For example, in wheat sister lines, investment in deeper root systems tended to be offset by reduced storage of water soluble carbohydrates in stems, the latter being important if sub‐soil water is unavailable.^41^ We highlight the context‐dependent effects of major drought‐adaptive traits on yield under water deficit.

### 4.1 Leaf area

**FIGURE 1:**
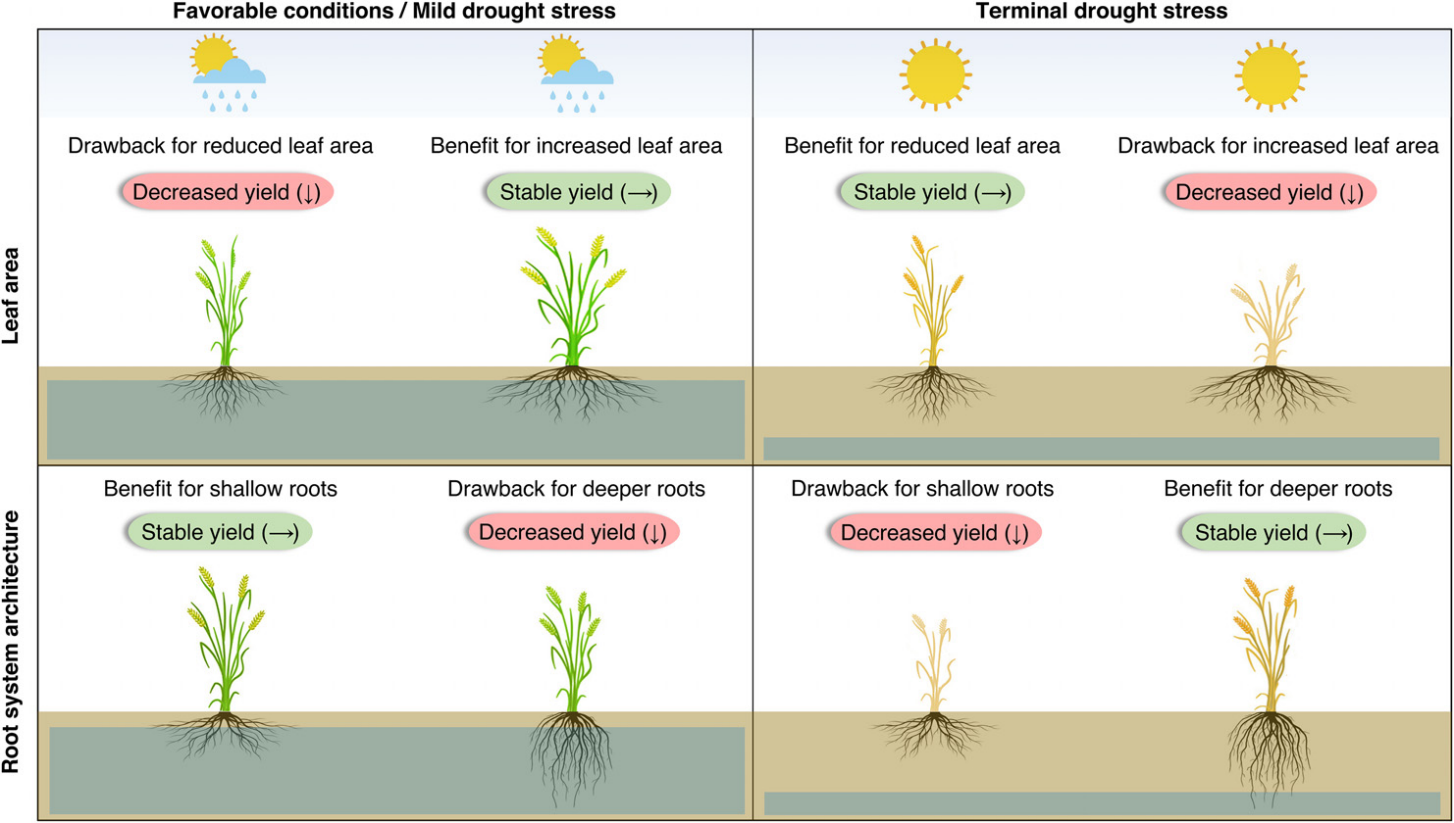
Yield benefits and drawbacks linked to leaf area and root system architecture under different drought scenarios. The most advantageous leaf and root phenotype is highly context‐dependent and impacted by several components, including climate, soil, or management. Reduced leaf area retains soil moisture and decreases hydraulic gradients, and is associated with yield reward under terminal severe stress; whereas, it lowers cumulative photosynthesis during the crop cycle and displays a yield penalty under favorable conditions/mild drought stress. Deeper roots increase water uptake from very deep soil layers and possess yield reward under terminal severe drought stress conditions where deep water is available; whereas, it leads to a lower nutrient uptake and is suboptimal under favorable conditions for nutrient foraging in upper soil layers

For environments that experience long and severe drought episodes, genotypes possessing a small leaf area or reduced transpiration have an advantage, as they retain soil water for later phases of the crop cycle for grain filling (Figure 1).^42^, ^43^ For example, a reduced transpirational leaf area in sorghum *Stg* near‐isogenic lines relative to their recurrent parent under severe water deficit enabled increased water extraction during grain filling, leading to better biomass production, grain number, and grain yield.^44^ Simulation modeling across a range of climatic scenarios and management practices suggests that reduced leaf area confers a yield reward under severe drought stress but negatively impacts crop yield and biomass accumulation under less severe circumstances in crops such as maize^45^ and sorghum.^46^ By contrast, under rainfed field conditions that usually experience sporadic drought patterns, the ability to maintain leaf area during soil water deficit is mainly responsible for determining crop yields (Figure 1). For instance, simulation modeling revealed that early vigor traits in wheat resulted in up to 16% yield advantage via genotypes possessing doubled early leaf size, at wetter sites or years.^47^ Importantly, the maintenance of leaf growth characteristics could confer four major benefits to the crop: (a) a higher photosynthesis at canopy scale, in particular during the pre‐flowering stage that affects grain number^48^; (b) a reduction in soil water evaporation, while facilitating efficient use of water via transpiration^47^; (c) a drop in leaf temperature due to higher transpiration rate^49^; and (d) a decrease in seed abortion rate caused by source‐sink relationships.^50^

### 4.2 Root architecture

A deep, wide‐spreading, and branched root system is beneficial for most crops grown in deep soils under moisture deficit conditions.^18^, ^51^ This notwithstanding, breeding programs focused on improving grain yield under drought have often resulted in the development of crop varieties with reduced root biomass.^52^, ^53^ This is mainly because, the spatial distribution of roots in the deep soil profile, but not root biomass or root length, governs the capability of root systems to efficiently uptake soil water. For instance, increased root distribution in deep soil layers (>30 cm soil depth) facilitated better water uptake and adaptation in drought tolerant chickpea genotypes.^54^ Therefore, longer and deeper roots with compact branching angles could be accompanied by high root length density in the deep soil profile to precisely capture water from the soil, which is dry at the surface but holds moisture in deeper layers (Figure 1). Soil nutrients such as phosphorous, potassium, iron, and manganese, are immobile and usually present in the upper soil layers. Hence, it should be considered that a substantial increase in root length density in the deep soil layers at the expense of upper layers would limit nutrient acquisition from the top soil and may be associated with crop yield penalties.^55^ By contrast, in shallow soils that receive intermittent rainfall during the crop growing season or under optimal soil moisture conditions, an extensive, broad and shallow root system is preferable for enhancing crop productivity (Figure 1).

### 4.3 Crop cycle duration

Drought escape is an adaptive mechanism that involves the rapid development of a plant to complete its entire life cycle prior to the onset of drought stress. Following the concept of drought escape, the duration of the crop cycle, which is mostly determined by genes affecting flowering time, also plays a critical role in enhancing productivity under water deficit. A long crop cycle is advantageous under favorable conditions, since it increases the interception of incident solar radiation, but is associated with a yield penalty under severe terminal drought, because it depletes soil water reserves before the end of the crop cycle.^7^ This was found to be true for wheat genotypes selected in Mediterranean‐type climates with frequent occurrences of terminal drought stress, where modern varieties with a drought escape strategy showed substantially higher production due to reduced risk of water stress during reproductive or grain filling stages.^56^, ^57^ The concurrent confounding effects of phenology‐related components, such as the duration of vegetative period, flowering time, and crop cycle as the major factors affecting water uptake, suggest that interactions of these traits with specific leaf or root ideotypes can be explored through a combination of experiments and modeling (Boxes 1 and 2).

Taken together, the substantial context‐dependency accompanied with above‐ and below‐ground plant traits, highlights the need for more targeted studies considering G × E × M interactions in crops to stabilize yields in fluctuating environments.^58^

BOX 1 **A probabilistic approach for drought adaptation**

A deeper understanding of the dual effects of most drought‐adaptive traits is crucial for tailoring future crops with a phenotype matching the target climatic scenarios. In theory, custom‐designed crops should carry a genetic predisposition for a particular leaf or root architecture together with mechanisms allowing growth plasticity in response to water deficit, as observed in wheat.^26^ This could significantly increase a crop's ability to maximize resource capture from the surrounding environment while reducing the impact associated with climatic fluctuations. The context‐dependency of nearly every trait on yield needs a two‐step approach: (a) identify the combination of alleles that influence the response of studied traits to environmental conditions, and (b) detect the response of yield to traits or allelic combinations in most frequent scenarios sensed by plants in a given region.^7^ To this end, Millet et al.^48^ suggested a simplified approach using a regressive model of responses to environmental conditions in maize, with genotype‐dependent sensitivities that were modeled by genomic prediction. This consisted of three major phases: (a) to establish response curves of yield components to soil water deficit, evaporative demand, and light in a multi‐site field experiment; (b) to simulate the sensitivity of each genotype based on genomic prediction; and (c) to predict yield of hundreds of genotypes in hundreds of fields in which drought stress differed between years. Because farmers do not know at the time of sowing the actual environmental scenario that will be sensed by plants, the choice of genotype will ultimately depend on the probability of environmental scenarios in each farmers' field,^45^ and the farmers' own choice between maximum gain versus risk avoidance. Such a probabilistic approach, which is based largely on the genetic variability of adaptive traits and on their context‐dependent effects, may eventually assist breeders to improve drought adaptation of major field‐grown crops.

BOX 2 **Glossary**

**ADAPTIVE TRAIT**

A phenotypic trait that differs with existing environmental scenarios for a particular genotype, and which can maximize fitness or production in specific environmental conditions.

**ANTHESIS**

The period during which a flower is completely open and functional.

**DROUGHT ADAPTATION**

The ability to sustain biomass production or crop yield, despite the occurrence of drought episodes during the crop cycle.

**ENVIRONMENTAL SCENARIO**

A clustered pattern of time courses for soil moisture status, evaporative demand, light, and temperature in diverse fields (a particular field can experience different scenarios in different years).

**EXPRESS EDIT**

A system that incorporates gene editing directly in the speed breeding system, and has the potential to bypass the bottlenecks of in vitro manipulation of plant materials.

**GENE REGULATORY NETWORK (GRN)**

A group of molecular factors (genes, RNA, and proteins) that interact directly or indirectly with each other and together influence a biological process of interest.

**GENETIC GAIN**

Improvement in the average genetic value in a population or in the average phenotypic value due to selection within a population over multiple cycles of breeding.

**GENOMIC BREEDING**

A breeding approach that uses “‐omics” data, knowledge resources, genes, and technologies, developed from genomics and genome editing research for crop improvement.

**HAPLOTYPES**

A group of alleles within an organism that are inherited together from a single parent.

**HAPLOTYPE‐BASED BREEDING**

A promising breeding approach for developing custom‐made crop varieties by introgressing superior haplotypes in elite breeding lines.

**HARVEST INDEX**

Harvest index is defined as the ratio of harvested grain to total shoot dry matter, and it can be used as a measure of reproductive efficiency.

**HYDRO‐PATTERNING**

A root developmental response where lateral roots preferentially initiate to the side in contact with water.

**HYDROTROPISM**

The directed growth of roots toward water or moisture gradients.

**HYPERSPECTRAL IMAGES**

Refers to the images in which one continuous spectrum is measured for each pixel.

**IDEOTYPE**

A biological model that is anticipated to perform in a predictable manner within a specific Environment.

**LEAF WATER POTENTIAL**

Indicates the whole plant water status and contributes to plant‐level physiological drought adaptation.

**LINKAGE DRAG**

The undesirable effects of deleterious alleles genetically associated with the desired trait.

**OSMOTIC ADJUSTMENT**

A reduction in osmotic potential attained by the accumulation of solutes in response to osmotic stress.

**OSMOLYTE BIOSYNTHESIS**

The synthesis and accumulation of diverse osmolytes in plants for combatting osmotic and oxidative stress.

**PHYLLOCHRON**

The time interval between the appearances of successive leaves on the main stem of the plant.

**PROTOSPACER ADJACENT MOTIF (PAM)**

The DNA motif flanking the target sequence that is indispensable for target recognition and cleavage by CRISPR‐Cas systems.

**SPEED BREEDING**

A breeding strategy that greatly shortens generation time in plants, by using supplemental lighting under glasshouse conditions and by extending the photoperiod to a day‐length of 22 hours.

**STOMATAL CONDUCTANCE**

A measure of the rate of carbon dioxide uptake and water loss (viz. transpiration) through the stomata of a leaf, as evaluated by the degree of stomatal aperture.

**SYSTEMS BIOLOGY**

A holistic approach for deciphering the complexity of biological systems that starts from the understanding that networks that form the whole of living organisms are more than the sum of their parts.

**TARGET POPULATION OF ENVIRONMENTS (TPE)**

The set of fields and future climate scenarios in which the crop varieties produced by a breeding program will be grown.

**TILLERING**

The production of lateral shoots by a plant, mostly a grass or cereal, from the base of the stem.

## 5 HARNESSING GERMPLASM DIVERSITY

A major constraint for tailoring crop varieties is the limited genetic diversity for key traits available in modern crop gene pools, mainly due to domestication and breeding bottlenecks.^59^ National and international gene banks contain a great source of diverse alleles and may hold the key to addressing this limitation. Three major categories of genetic resources can be explored, namely crop wild relatives, secluded gene pools (eg, landraces), and modern breeding lines.

Evaluating large germplasm collections for identifying variation in drought‐adaptive traits may not always be feasible. In such cases, selecting an economically feasible set of accessions with a higher probability of capturing beneficial allelic variation is much more preferable.^60^ The Focused Identification of Germplasm Strategy (FIGS) was used to enhance the efficiency of detecting specific drought‐adaptive traits from faba bean germplasm collections.^61^ FIGS utilizes agro‐ecological data to generate a priori information, which is then used to identify a group of accessions possessing the desired adaptive traits. An impressive illustration of this strategy came from a recent field evaluation of FIGS wheat panels prioritized based on tolerance to drought stress, which revealed over 45% of lines having greater plant biomass under drought than the adapted check varieties.^62^ With better access to the genetic variability found in natural populations of wild relatives and landraces, the door is open for retrieving drought‐adaptive traits; several of which are encoded by alleles that disappeared in domesticated crops, or which evolved individually in diverse crop lineages.^60^

Next‐generation sequencing and high‐throughput genotyping platforms can be used to characterize allelic diversity in genetic resources,^63^ and suitable lines carrying desired combinations of alleles can be used to develop specific leaf and/or root ideotypes for target environments.^64^ Recent sequencing efforts, including The 3000 Rice Genome^65^ and The 3000 Chickpea Genome^66^ hold promise to provide novel insights into intra‐species genetic variation and evolutionary crop history. The integration of gene bank passport data and weather data from the target population of environments (TPE) can be utilized to identify superior haplotypes for specific adaptive traits, which could be used in haplotype‐based breeding (discussed below).^11^ Additionally, deleterious alleles (genetic load) associated with the trait(s) of interest could be identified by utilizing genomic evolution parameters and amino acid conservation modeling, as demonstrated in cassava^67^ and chickpea,^66^ and eliminated using molecular breeding or genome editing strategies.^68^ As a result, superior parental lines containing preferred alleles at each locus and with minimum undesirable genetic load could be identified and integrated into breeding programs to tailor crops with desired allelic combinations.

## 6 INNOVATIVE GENOMIC BREEDING STRATEGIES

Given the enormous genetic diversity available in germplasm repositories, extracting meaningful information from these resources require new‐age breeding strategies. Genomic breeding plays a significant role in crop improvement,^69^ as highlighted by the development of a large number of improved field‐grown crops with better adaptation to drought conditions (Table 1). Some genomic breeding approaches are being successfully used in major crops such as rice, wheat, maize, etc. in the highly industrialized world. However, crops grown in marginal environments such as pearl millet, sorghum, chickpea, pigeonpea, and cassava remain largely eluded from this success. Drought and desertification in the dryland regions result in an estimated loss of 12 million hectares of land every year, which accounts further in a loss of 20 million tons of food grain production.^84^ This demands an urgent investment in improving the drought adaptation of dryland crops using modern technologies, for ensuring future food security. Here, we describe how genomic innovations coupled with modern breeding efforts offer opportunities for designing drought‐adaptive crops across the world, particularly in South Asia and Sub‐Saharan Africa.

**TABLE 1:** Key success stories of genomic breeding strategies to improve drought adaptation and grain yield in field‐grown crops

Strategy	Crop species	Target QTL/gene	Yield in WW	Yield in WS	Growth/physiology	Reference(s)
Marker‐assisted backcrossing	Wheat	*Qyld.csdh.7AL*	↑ Grain yield	↑ Grain yield	↓ Canopy temperature	^.^ ^70^
			↑ 1000‐grain weight	↑ Tiller number	↓ Stress‐sensitivity index	
				↑ 1000‐grain weight		
				↑ Biomass yield		
	Sorghum	*Stg1‐4* QTLs	=	↑ Grain yield	↓ Green leaf area at anthesis	^15^, ^44^
				↓ Tiller number	↓ Canopy size at anthesis	
					↑ Post‐anthesis water use	
					↓ Root angle	
	Pearl millet	*LG02‐QTL*	NA	↑ Grain yield	↓ Transpiration rate	^71^, ^72^
				↑ Panicle harvest index	↓ FTSW threshold	
				↑ Biomass yield	↑ Leaf ABA content	
					↓ Transpiration at high VPD	
					↑ Root growth in deeper soil	
	Chickpea	*QTL‐hotspot*	NA	↑ Grain yield	↑ Root length density	^73^, ^74^
				↑ 100‐seed weight	↑ Root dry weight	
					↑ Rooting depth	
Marker‐assisted gene pyramiding	Rice	*qDTY*_*3.2*_ and *qDTY*_*12.1*_	↑ Grain yield	↑ Grain yield	↓ Days to flowering	^.^ ^75^
					↓ Plant height	
	Rice	*qDTY*_*2.2*_ *, qDTY*_*3.1*_ and *qDTY*_*12.1*_	↑ Grain yield	↑ Grain yield	↓ Plant height	^76^
					↓ Days to flowering	
Marker‐assisted recurrent selection	Maize	Multiple QTLs	↑ Grain yield	↑ Grain yield	NA	^77^
	Maize	Multiple QTLs	↑ Grain yield	↑ Grain yield	↓ Anthesis silking interval	^78^
					↑ Plant height	
Genomic selection	Maize	Multiple QTLs	↑ Grain yield	↑ Grain yield	↓ Anthesis silking interval	^79^
	Maize	Multiple QTLs	↑ Grain yield	↑ Grain yield	↓ Transpiration at high VPD	^80^
					↓ Anthesis silking interval	
	Maize	Multiple QTLs	↑ Grain yield	↑ Grain yield	NA	^81^
Genome editing	Maize	*ZmARGOS8*	=	↑ Grain yield	↑ Plant height	^13^
					↑ Ear height	
	Tomato	*SlLBD40*	NA	NA	↓ Water loss	^14^
					↓ Leaf stomatal conductance	
					↓ MDA content	
					↑ Fv/Fm ratio	
					↑ Leaf water potential	
	Rice	*OsDST*	NA	NA	↑ Leaf width	^82^
					↓ Stomatal density	
					↑ Leaf water retention	
	Soybean	*GmDrb2a* and *GmDrb2b*	NA	NA	↑ Drought tolerance	^83^

Abbreviations: ABA, abscisic acid; FTSW, fraction of transpirable soil water; MDA, malondialdehyde; NA, not available; QTL, quantitative trait locus; VPD, vapor pressure deficit; WS, water‐stress conditions; WW, well‐watered conditions. ↑, increased; =, maintained; ↓, decreased.

### 6.1 Haplotype‐based breeding

Plant breeding depends on recombination to combine preferred combinations of traits for developing improved crop varieties. Traditional breeding uses the heritability of phenotypes as a key criterion to evaluate genetic combinations. The genomic regions defined by quantitative trait loci (QTLs) typically contain multiple candidate genes and genetic variations, which either do not confer desired traits or possess harmful effects. Combinations of genomic loci that contribute to the preferred phenotype can be considered as groups of haplotypes that are determined by underlying genetic variation. Use of such genomic information enables the breeder to select superior haplotypes for designing ideal crop varieties in silico and deploy them in breeding programs. We refer to this concept as haplotype‐based breeding, representing the evolution and a much more accurate version of “breeding by design”.^85^

The retrospective and prospective approaches suggested by Bevan et al.^11^ hold great potential for deploying a haplotype‐based breeding strategy to develop drought‐adaptive crops. In the retrospective approach, the genomic regions and the underlying haplotypes preferred by breeders over time can be identified by sequencing the genomes of important breeding lines, which have been widely evaluated across multiple environments and years in past decades. This will provide an overview of the breeders' selection decisions over time and help determine superior haplotypes related to previous breeding success. This approach was demonstrated recently by identification of candidate genes and signatures of artificial selection associated with seed size and weight using sequencing data for 200 accessions of cultivated flax (*Linum usitatissimum* L.).^86^ Furthermore, the information acquired by employing a retrospective approach can be used to identify the function of genomic regions containing the haplotypes, referred to as haplotigs,^87^ and determine the underlying desired and deleterious alleles associated with adaptive traits. By contrast, a prospective approach can be utilized by sequencing large ancestral populations and undomesticated crop varieties, to determine conserved inherited haplotigs with huge genetic variation and to identify combinations of superior haplotypes. This approach was used in a recent study to detect superior haplotypes for key genes related to drought tolerance component traits, which can be deployed in haplotype‐based breeding of pigeonpea.^88^

**FIGURE 2:**
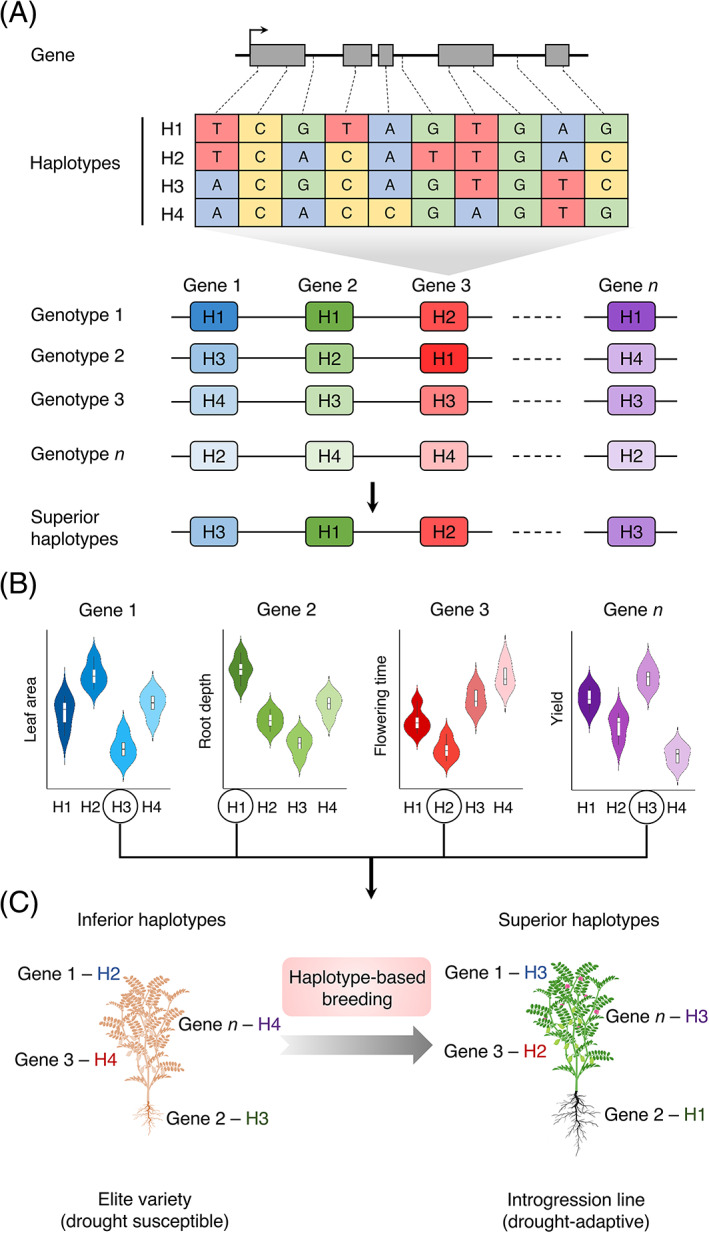
Haplotype‐based breeding scheme for designing drought‐adaptive crops. (A) An example of haplotypic variation (H1‐H4) underlying a particular gene (gene 3) on a chromosome. A combination of various haplotypes for different genes (gene 1, gene 2, gene 3, gene *n* …) has been shown in multiple genotypes (genotype 1, genotype 2, genotype 3, and genotype *n*). Superior haplotypes are selected for various genes based on the preferred combinations of phenotypes, expressed by the respective haplotype. (B) Difference in the performance of four traits associated with drought adaptation that are influenced by genetic variation in gene 1 (leaf area), gene 2 (root depth), gene 3 (time to flower) and gene *n* (yield), is illustrated using violin plots. Analysis of variance results indicate that H3 is the superior haplotype for gene 1, H1 for gene 2, H2 for gene 3, and H3 for gene *n*. (C) Introgression of superior haplotypes for multiple adaptive traits in an elite variety using haplotype‐based breeding approach will enable development of an introgression line possessing improved drought adaptation

Haplotype‐based breeding represents a promising strategy for crops in which large germplasm collections are characterized both at the sequencing and phenotyping level. Assembling desired haplotype combinations in elite crop varieties will enable informed decision‐making in breeding programs.^69^ For instance, a combination of superior haplotypes of previously validated genes that confer small leaf area, deeper root system, early flowering, and higher yield can be used to design crop ideotypes for improved adaptation to terminal drought stress (Figure 2). Taken together, haplotype‐based breeding is expected to design future crops with desired adaptive traits, while demanding less monetary investment and in the absence of challenging public acceptance.

### 6.2 Genome editing

Introgression of desired traits into an elite variety is often impaired by the random nature of recombination and linkage drag, making conventional breeding a time‐consuming and laborious process. Heavy dependence on natural or random genetic diversity is a major constraint delaying the breeding process and leading to an unpredictable outcome.^89^ In contrast, genome editing holds enormous potential to generate precise, efficient, and targeted alterations in crop plants. It can be performed with any crop, including those that possess complex genome architecture and are not readily bred using conventional approaches.^90^ The recent development of clustered regularly interspaced short palindromic repeats (CRISPR)‐CRISPR‐associated nuclease protein (Cas) systems have brought genome editing into the limelight.^13^, ^91^ Several gene knockout, insertion or replacement mutants are developed by CRISPR‐Cas9‐mediated editing in field‐grown crops to improve their drought adaptation characteristics (Table 1).

**FIGURE 3:**
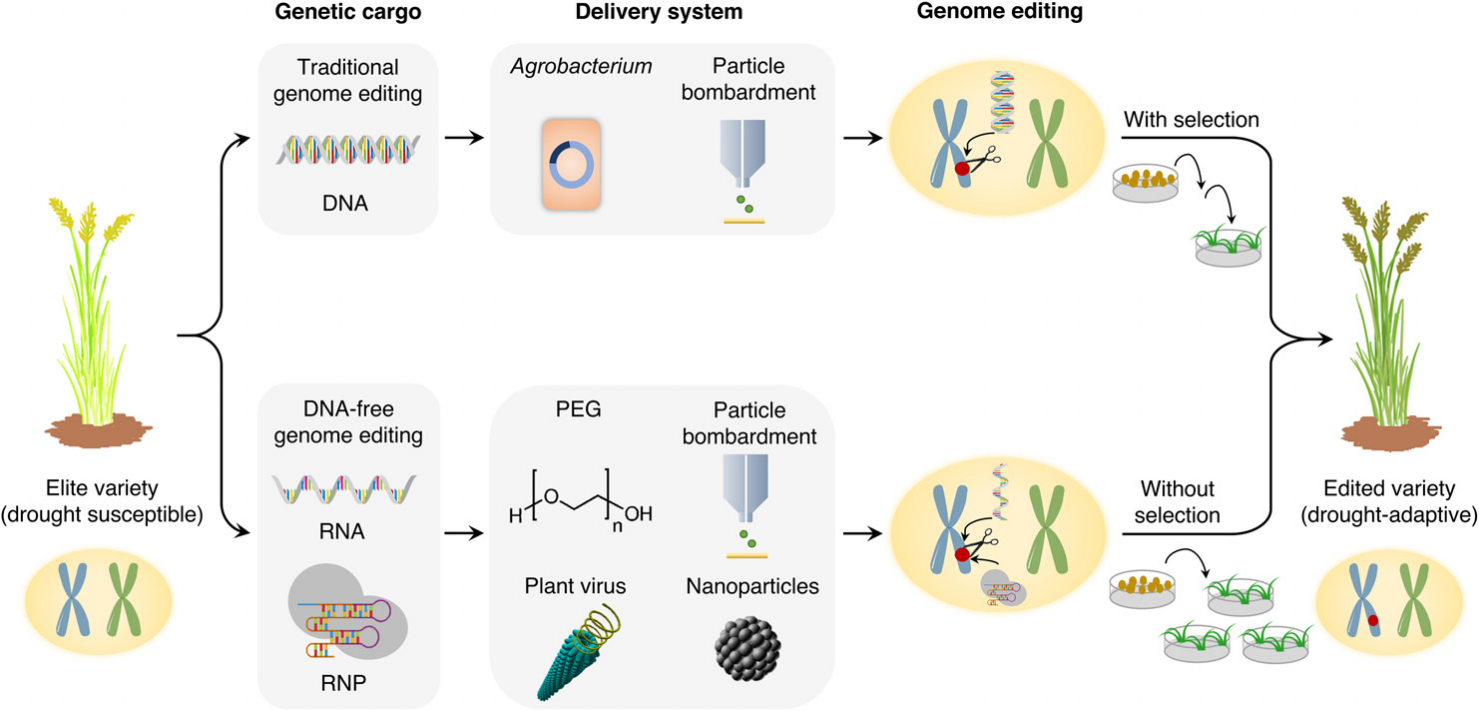
Strategies for delivery of CRISPR‐Cas systems to plants. Traditional delivery systems for genome editing include CRISPR‐Cas DNA together with selection pressure. Genetic segregation via selfing and crossing results in the development of transgene‐free plants. Transient delivery methods for DNA‐free genome editing involves the use of CRISPR‐Cas reagents, such as RNA and ribonucleoproteins (RNPs). CRISPR‐Cas reagents degrade after transient expression, and the edited plants can be regenerated without applying any selection pressure. DNA, deoxyribonucleic acid; mRNA, messenger RNA; RNP, ribonucleoprotein; PEG, polyethylene glycol

Technical breakthroughs in the genome editing toolkit provide opportunities to exploit mutations giving rise to optimal shoot and/or root architecture for designing crops for the future. CRISPR‐Cas9‐mediated base editing is one such example, which can precisely change one DNA base into another in the absence of a DNA repair template.^92^, ^93^ Recent development of a PAM‐less CRISPR‐SpRY toolbox, which disrupts a PAM restriction barrier of targeting only GC‐rich DNA regions, has greatly expanded base editing scope in crops.^94^ Traditional transgene‐mediated CRISPR‐Cas delivery techniques (Figure 3) may be linked with undesirable genetic changes,^95^ with extended breeding cycles and regulatory constraints. Therefore, ribonucleoprotein (RNP)‐based DNA‐free genome editing^96^ is considered a major leap toward developing genome‐edited crops with a lower risk of undesired off‐target modifications, and satisfying present and future agricultural needs from a regulatory perspective. This technology has been accomplished in grapevine and apple through protoplast transformation,^97^ in bread wheat via in planta particle bombardment,^98^ and in tobacco by virus infection^99^ (Figure 3). The newly emerged CRISPR‐Cas12a^100^ (formerly Cpf1) and Cas12b^101^ (formerly C2c1) systems have several key advantages over Cas9, such as preferring T‐rich PAMs (enabling alternative targeting sites to Cas9), and generating staggered ends of DNA double stranded breaks as opposed to blunt ends created by Cas9. Hence, the application of CRISPR‐Cas12a and Cas12b could provide attractive alternatives to CRISPR‐Cas9 systems in designing crop ideotypes for specific drought environments.

Drought adaptation processes in crops are often regulated by complex genetic mechanisms with the combined expression of numerous genes. Based on Golden Gate and Gateway assemblies, multiplex CRISPR‐Cas expression systems have been developed for different applications in plants.^102^ For example, multiplex CRISPR‐Cas systems have been used for generating quantitative trait variation in tomato by editing cis‐regulatory elements in the promoter^103^ and for de novo crop domestication by simultaneous editing of multiple trait genes in wild tomato.^104^ Furthermore, a fusion of diverse effector domains to catalytically inactivated Cas (dCas) proteins has repurposed CRISPR systems for transcriptional regulation and epigenome editing in plants.^105^ Such innovative genome editing strategies could be harnessed to tailor shoot and root architectural traits in custom‐designed crops. Moreover, the integration of speed breeding (see later) with genome editing technology, referred to as “Express Edit,”^106^ will enable genome editing to bypass the restrictions imposed by in vitro manipulation of plant tissues. This, in turn, will facilitate the inclusion of genome editing into large‐scale breeding programs for crop improvement.

### 6.3 Systems biology‐based breeding

Drought adaptation is a complex trait comprising an intricate regulatory network of phytohormones, transcription factors, and kinases. It is essential to further unfold the direct interactions between these key players and their downstream targets to shortlist most potential candidates for breeding purposes. This demands a systems biology approach to unravel the temporal dynamics and spatial configurations specifying the biological phenomenon of interest.

In plants, cellular processes are typically governed by gene regulatory networks (GRNs) that can influence a trait of agronomic interest.^107^ To design specific crop ideotypes, GRNs can identify the most promising candidate genes, predict the network behavior arising from the altered genes and the resulting phenotypes of the traits modulated by the network. For instance, a limited number of QTLs and candidate genes regulating leaf senescence, an important determinant of drought adaptation, have been identified till date in crops such as wheat.^108^, ^109^, ^110^, ^111^ A comprehensive understanding of the network of genes modulating this process may facilitate the development of wheat varieties having a senescence profile tailored to augment nutrient remobilization, while enabling improved yield and adaptation to drought conditions. In a recent study, analyses of gene expression data and GRN modeling led to the identification of key transcriptional regulators (such as *NAM‐A2*) that coordinate flag leaf senescence in wheat.^112^ Such approaches will help to understand network performance and identify breeding targets that are usually not detected by traditional forward‐ and reverse‐genetics strategies, and utilize them to manipulate an entire network to build the desired phenotype. Novel insights into the complex associations existing between different adaptive traits and their corresponding genes at the systems level will help to identify preferable haplotypes and to design haplotype‐based breeding scheme. In rice, meta‐expression analysis together with co‐expression network offered insights into the function of multiple genes and their interactions at a systems level, which in turn helped in the selection of desired haplotypes for haplotype‐based breeding.^12^ Furthermore, dynamic modeling and virtual mutations have shown promise in determining GRN engineering targets in order to tailor the desired phenotype.^113^ Therefore, upcoming efforts to determine molecular breeding targets should focus on such candidates, and assess the impact of virtual mutations in silico by modifying the network model and capturing transient interactions among the GRN.^114^ For drought adaptation in crops, such dynamic models will offer robust support for validating the hypothesis obtained from field experiments and accurately defining technologies for rationalizing breeding strategies.

A systems biology approach will provide hints to detect genetic regulators exercising the largest effect on GRNs, even where gene redundancies, mild‐effect genes, or feedback loops hinder traditional gene investigation capabilities.^112^ This approach will be particularly relevant for designing ideotypes for crops with complex genomes and multigenic traits, where systems‐scale strategies are predicted to outperform the restrictions of conventional breeding approaches in the context of efficacy and speed.

### 6.4 Genomic selection and speed breeding

**FIGURE 4:**
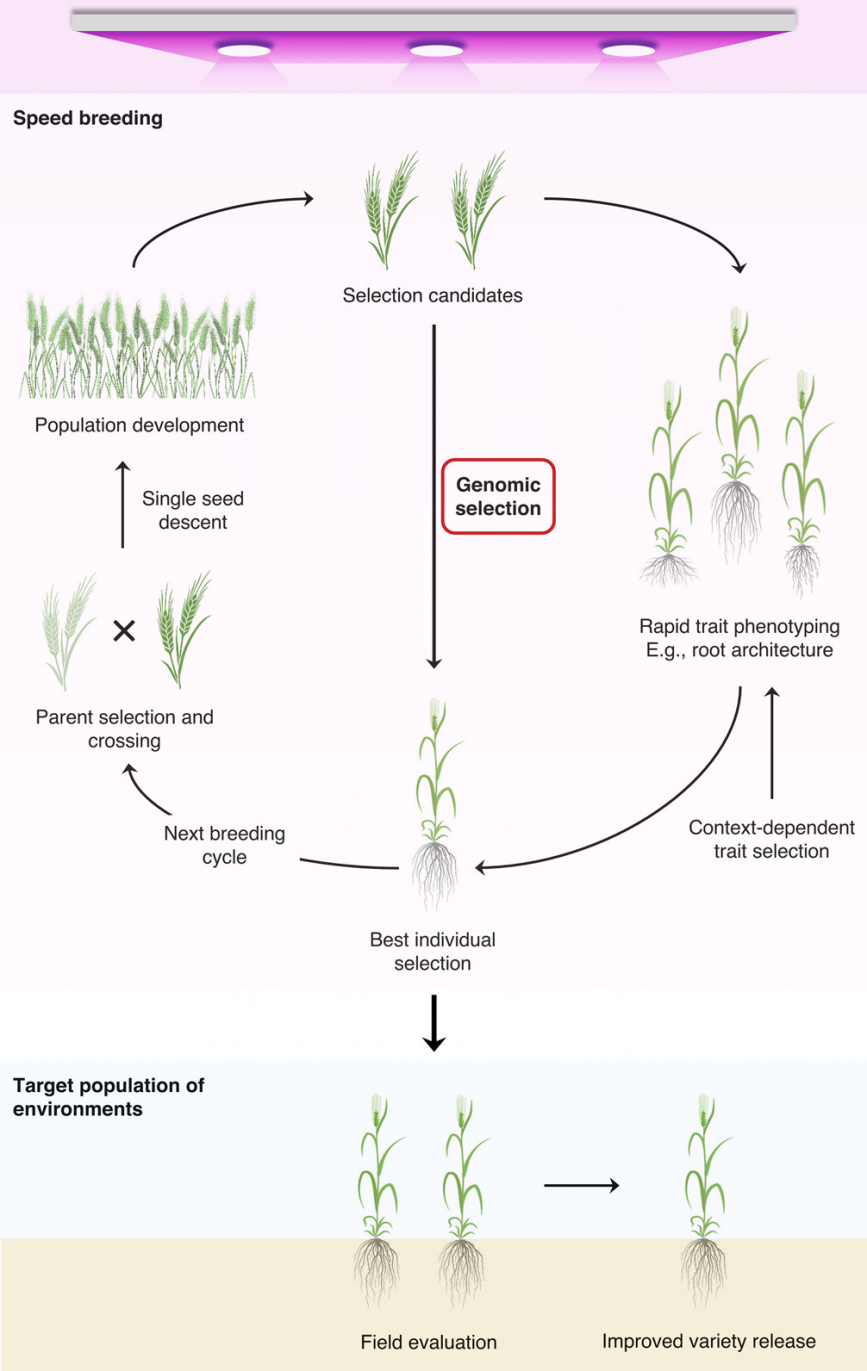
Integrating genomic selection and speed breeding to fast‐forward genetic gain in crops. The breeding cycle length decreased by genomic selection could be further reduced by performing population development under speed breeding conditions. Selection candidates could be phenotyped for grain yield secondary traits (eg, root architecture) in the glasshouse, and plants that carry the desired trait could be selected. Context‐dependent selection of the trait will enable plants to be better adapted to the target environment prior to selecting more complex traits, such as yield. Phenotyping and selecting plants under speed breeding in the glasshouse could further improve selection intensity and the rate of genetic gain

Innovations in genomic technologies are of particular relevance for improving future crops. However, it takes multiple years for an improved crop variety to be advanced and released for commercial cultivation due to prolonged breeding cycles, which in turn hampers the gain in productivity. Genomic selection and speed breeding approaches are crucial to address the long breeding cycle issue in crops. Genomic selection, which estimates the genetic merit of breeding lines for complex traits such as drought adaptation and increases the efficiency of selection process, is being successfully deployed in many crop breeding programs (Table 1). A good illustration of its impact came from a recent field evaluation of drought tolerant maize hybrids (called as “AQUAmax” hybrids), which possessed substantially higher yields under both optimal and drought stress conditions.^80^, ^115^ On the other hand, speed breeding by achieving up to six generations per year for wheat, barley, chickpea, and pea using specific and highly controlled environmental conditions such as 22 hour‐long photoperiods, has emerged as a popular approach for accelerated crop development.^116^ For custom‐designed crops, genomic selection can save time and resources typically for traits that are phenotyped during the final stages of the variety development process and those that are costly to measure, for example, yield. While speed breeding can decrease generation times drastically, the genetic gain associated with this technique can be further improved by applying genomic selection at every generation to choose parents for the next generation (Figure 4). The strategy of combining speed breeding with genomic selection, referred to as “speed GS,” holds potential for fast‐forwarding the rate of genetic gain in crop improvement.

A particular haplotype with the highest genomic estimated breeding value can be determined for each genomic region and demarcated by linkage disequilibrium blocks. Desired haplotypes can then be stacked in a cropping line by using an optimum array of crosses. This approach of combining genomic selection with superior haplotypes, called “haplo‐GS,” can be integrated with speed breeding to rapidly tailor crop varieties with high performance across multiple adaptive traits. The haplo‐GS approach could more precisely illustrate the complex relationships between genotype and phenotype, relative to individual SNPs, ultimately enhancing selection gain per unit of time.

## 7 CONCLUSIONS AND PERSPECTIVES

Future food security will depend on the continuous development of improved crop varieties, which sustain greater yields with minimum agronomic inputs and are better adapted to climate change. Crops can encounter fluctuating drought scenarios, ranging from mild to severe drought episodes at the beginning, mid, or toward the end of the crop cycle. Attributes such as soil depth and constitution, water availability, climate, and management practices also impact crop responses to water deficit. As a result, conceptualizing a drought‐adaptive ideotype optimized for an array of scenarios may not be possible, but distinct traits related to drought adaptation that are similar across species grown under diverse field conditions could be used as targets for custom‐designing crops for specific environments. Developing such designer crops that integrate individually strengthened leaf and root systems, can be simplified by implementing recent technical breakthroughs in crop modeling, second and third‐generation sequencing, novel breeding techniques, genome editing, deep learning approaches, and high‐throughput phenotyping.

Combining genetic resources and transformative capabilities, ranging from genomic breeding to synthetic biology, will be essential for tailoring crops that improve food security and decrease the impact of agriculture on the environment. Notably, the integration of artificial intelligence (AI) and machine learning algorithms into the processing of hyperspectral images and weather data will enable early‐stage prediction of drought scenarios. In turn, the AI system will allow farmers to make more informed decisions at every step of the crop production cycle. Finally, the context‐dependent effect of each trait, multiplicity of combined traits, and genotype × environment interactions for each trait necessitates the use of modeling, to predict the effect of thousands of allele combinations in thousands of fields. This will help to derive a probabilistic approach for identifying the most desirable allelic combinations in a given field/region depending on the scale of the target environment.

A custom‐designed crop will be a valuable asset in our attempts to quench agriculture's growing thirst, and maximize productivity while enhancing yield stability in the face of enhancing environmental fluctuations. This ambitious aim needs the collaborative will and efforts of breeders, geneticists, physiologists, systems modelers, and bioinformaticians alike.

## CONFLICT OF INTEREST

The authors declare no competing interests.

## AUTHOR CONTRIBUTIONS

R.K.V. conceived the idea; R.K.V. and R.B. wrote the manuscript. All authors substantially contributed to the discussion of content, and reviewed and/or edited the manuscript before submission.
